# Microbial Ecotoxicology—40 Years on

**DOI:** 10.3390/life15040514

**Published:** 2025-03-21

**Authors:** Tim Ford

**Affiliations:** Department of Biomedical and Nutritional Sciences, University of Massachusetts Lowell, Lowell, MA 01854-5125, USA; timothy_ford@uml.edu

**Keywords:** microbial ecotoxicology, indicators, environmental stress, microbial activity, gene expression

## Abstract

Although ecotoxicology was emerging as a field through the 1970s, the incorporation of microbial indicators into the framework has been slower to evolve. The exploration of microbes as sensitive toxicity tests began in the late 70s and early 80s (with the emergence of Microtox^®^ and other simple tests). However, the applications have been limited, beyond water and wastewater screening. This opinion piece reflects my own perspective on the field—from my early excitement in the 1990s for its possibilities, to a sense of frustration at the slow pace of new development and applications in the field—despite the surge of “omics” options. While microbiology still fails to lead the field of ecotoxicology, the potential remains.

## 1. Introductory Thoughts

The study of the toxic effects of chemicals on ecosystems is critically important in defining measurable endpoints for monitoring ecosystem health [1,2]. If we can do this at the microbial level, we can have rapid, actionable data to guide our decisions on the need for more advanced testing and remediation options. Microbes make sense; they grow fast and adapt rapidly to their environments [3]. In contrast, higher organisms used in the more traditional ecotoxicological approaches, e.g., bivalves, fish [4,5], may take far longer to adapt. I would argue that the potential for microbial ecotoxicology is there, but, to date, that potential has yet to be realized.

It seems extraordinary that it has been 40 years since I first started working on metal–microbe interactions [6,7] as a postdoctoral Fellow at Harvard University. Within a few years, that initial interest turned to applications in human health and, in particular, environmental health. The Napa Conference on Genetic and Molecular Ecotoxicology, held 12-15 October 1993 in Yountville, California [8], was probably one of the first times I talked about microbial ecotoxicology and the use of microbes as biomarkers of environmental stress. The context was an EPA-designated Superfund site, New Bedford Harbor (NBH), that has a long legacy of sediment contamination dating back to the 1800s and before. NBH is particularly known for industrial discharges of poly-chlorinated biphenyls and toxic metals by manufacturing companies from the early 1940s to the 1970s [9]. As part of Harvard’s NIH-funded superfund program, I was supported to study the microbial ecology of these contaminated sediments and, in particular, to look for microbial diversity indicators that reflected gradients of contamination from the inner harbor out into Buzzards Bay.

At the time, metrics of diversity ranged from simple indices that reflected species composition and abundance to more sophisticated indices at the molecular level. We began by using standard plate culture techniques using marine agar to determine surface sediment bacterial diversity using the Shannon–Weaver Diversity Index [10]. This is a simple mathematical tool to look at the number of species present (different microbial colonies) and their relative abundance. We actually had mixed results with this approach, with some studies showing lower microbial diversity with increased metal enrichment and others showing greater diversity associated with contaminated sites [11,12]. Of course, a major limitation of this approach is the fact that only a very small percentage of microbes will grow on a marine agar plate [13].

In 1999, we published a study on using “16S rRNA Restriction Fragment Length Polymorphism (RFLP) Analysis of Bacterial Diversity as a Biomarker of Ecological Health in Polluted Sediments from New Bedford Harbor, Massachusetts, USA” [14]. RFLP is defined by the National Library of Medicine as “a difference in homologous DNA sequences that can be detected by the presence of fragments of different lengths after digestion of the DNA samples in question with specific restriction endonucleases” [15]. Simply put, we cut up the DNA from bacterial communities along a pollution gradient and get different patterns as a result of chemical (or other) stress. A major limitation of this approach is that it is a very crude measure of microbial diversity and provides no information on physiological responses or what stressors are causing changes in DNA sequences. These could be chemical, physical or simply population changes along a gradient due to nutrient fluctuations [16]. To be fair, this was early on in the evolution of molecular techniques designed to look at microbial diversity and gene expression in response to pollutant exposure. The paper looked at DNA extracted from surficial sediments along a transect from the polluted inner harbor out into Buzzards Bay. RFLP allowed us to demonstrate higher bacterial diversity in NBH relative to Buzzards Bay but with what appeared to be greater genetic relatedness, suggestive of a more constrained ecological niche due to high levels of toxic contaminants. The apparent increased diversity of these microbial communities is in contrast to other studies that demonstrated that both taxonomic and genetic diversity of microbial communities declined in polluted environments, although these studies concluded that surviving microbial communities were well adapted to these stressed environments [17].

Although many studies in the 1980s and 90s provided contradictory evidence on pollutant effects on species diversity, we were very much of the mindset that natural microbial communities could be sensitive biomarkers of environmental stress, due to their rapid adaptations to change. Throughout the 1980s, there was increasing work on the adaptation of microbes to environmental stress [18,19,20] and on the role they might play in the fate, transport and transformation of pollutant chemicals [7,21]

A logical next step was to look for genes that might be expressed in response to toxic chemicals, for example, metal resistance genes in the presence of toxic metals and catabolic genes for organic contaminants. Alternatively, genes involved in pathways related to oxidative stress or detoxification might be up-regulated. Certainly, the Cytochrome P450 detoxification enzymes are used extensively as biomarkers in higher organisms [22]. Our initial focus in NBH was on the *ars* operon, on the basis that genes that convey arsenic (As) resistance had been well characterized in the literature. We were able to isolate both aerobic and anaerobic bacteria from shallow contaminated NBH sediments (18 µg/g As, dry weight) and grow them in marine agar supplemented with different concentrations of As (as sodium arsenate). Primer sets for three key genes (*ars A*, *B* and *C*) were then developed and the genes amplified to demonstrate presence in most of the isolates on both plasmids and genomic DNA [23]. We had demonstrated “proof of concept” in terms of presence of this arsenic resistance system but recognized that presence alone does not indicate exposure to bioavailable contaminants.

A move to Montana State University resulted in a shift in focus towards the Clark Fork River Superfund site (CFR). The CFR had for a long time been subject to contamination from toxic metals released by the Anaconda smelter that processed copper from the nearby town of Butte (resulting in the infamous Berkeley Pit). The work focused on the genes involved in metal detoxification as well as general stress genes that have been previously identified in *Pseudomonas aeruginosa*, a well-characterized bacterium and one that was isolated from all sites in CFR. The study found that the highest incidence of these candidate genes occurred at the most contaminated sites [24]. In a later study, and consistent with our earlier work in NBH [14], the most contaminated sites showed higher community activity, diversity and richness than reference sites (determined using dehydrogenase activity and denaturant gradient gel electrophoresis) [25]. Together with the earlier work in New Bedford Harbor, it is an important finding that increased microbial diversity, together with increased genetic relatedness, may be present at a polluted site. While perhaps counter intuitive—you expect microbes to be restricted by the presence of toxic chemicals—the adaptive abilities of microbes should never be underestimated. It also underscores the fact that diversity indices alone may not provide a good biomarker of a contaminated site, and it is necessary to take a more functional approach, which has now become possible with the evolution of a wide range of omics technologies (see Omics Section and Table 1).

**Figure 1:**
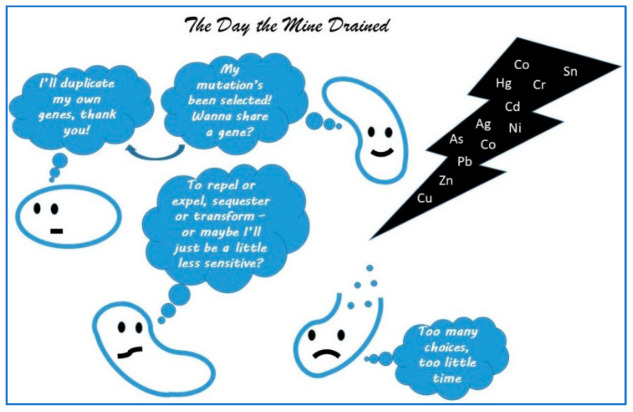
The Day the Mine Drained—a simplistic schematic redrawn from my special topic in Cells on “microbe-heavy metal interactions: the role of the resistome”, demonstrating in cartoon form some of the ways microbes react to the presence of heavy metals.

At this point, it may be useful to remind ourselves of the basic premise of ecotoxicology and where microbes may fit in. In 2022, I proposed a special topic in *Cells* on “microbe-heavy metal interactions: the role of the resistome”. While still open, the interest has been minimal, and I think the subject too specific when there are so many options for publication these days. The premise of the special issue was to attract papers that would address the complexities of the adaptive mechanisms of these microbes to a toxic environment—these mechanisms can fundamentally serve as the basis for microbial ecotoxicology. There are a multitude of ways in which microbes adapt, but the primary drivers that have been well-described in the literature are natural selection, horizontal gene transfer and gene duplication [26]. These mechanisms still need to be defined for specific systems and stressors to be useful in ecotoxicology—in addition to significant implications for bioextraction and bioremediation. I used a simplistic graphic, which I include here as Figure 1 to emphasize these mechanisms.

**Figure 2:**
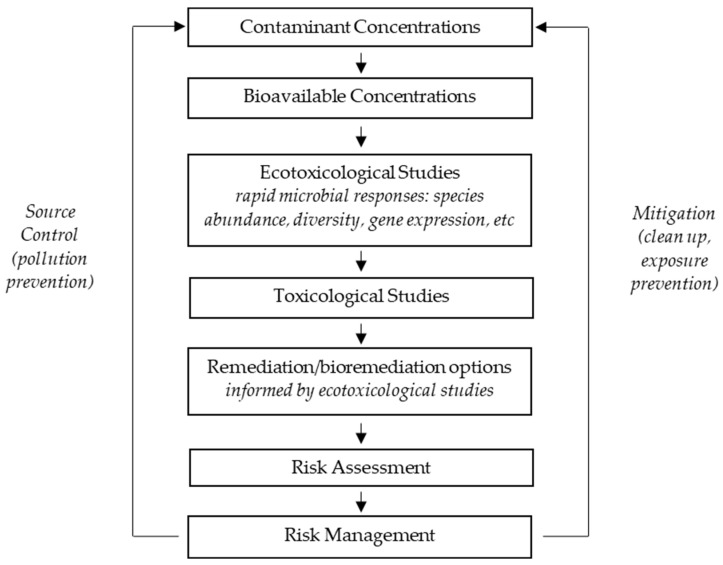
Scheme for risk management.

Figure 2 shows a simple scheme for risk management adapted from Cheng and Ford, 2009 [27], reflecting critical roles for microbes and microbial activity in both ecotoxicological studies and in the remediation processes. It is not surprising that microbial ecotoxicology is often discussed at conferences focused on pollution control, given that microbial responses to pollutants often inform us on the mechanisms that may be useful in bioremediation [27,28].

A number of conferences have been held in recent years, but it is difficult to assess their impact on the field. The following is a brief summation of those conferences.

## 2. Some Conferences of Note

As a result of the visionary leadership of the late Professor Shupei Cheng of Nanjing University, I was involved in organizing a series of international conferences on environmental health and pollution control (EHPC). Two of these conferences resulted in special editions in the journal *Ecotoxicology* in 2009 and 2011. The focus of the first edition, based on EHPC2008, was on genetic markers of contamination, ecotoxicological models, use of mouse models, degradative processes and ecological risk [27]; the second edition, based on EHPC2010, focused on contaminant concentrations, risk assessment, toxicity testing and treatment, monitoring and control [28]. Two papers are of particular note in relation to microbial ecotoxicology from these special editions. The first is a paper utilizing phospholipid fatty acid profiles of microbial communities as indicators of changing microbial composition in response to varying organic substrates [29], and the second is the use of biofilms (structural and functional attributes) as indicators of macrophyte-dominated lake health [30].

Two meetings were held in France on the subject of microbial ecotoxicology in 2013 and 2014. The report on these conferences [31] shows a strong interest in the topic but does not describe specific advances in the field. The website that was set up from these meetings is no longer active. Interestingly, the report concludes that, “We also strongly encourage authors of publications in the field to use the term “microbial ecotoxicology” as keyword, to increase the visibility of the international community of microbial ecotoxicologists”.

This is a clear reflection of a slowly moving field. A search of Pubmed on the term “microbial ecotoxicology” produced 93 citations, with the first two citations in late December 2014—the report on the meeting in France cited above [31] and a proposal for “geomicrobial ecotoxicology as a new subject in environmental sciences” [32]. This latter editorial, authored by Drs Ji-Dong Gu and Yanxin Wang resulted from the First International Conference on Geomicrobial Ecotoxicology, held Wuhan, P.R. China, in 2011.

A special issue of *Environmental Science and Pollution Research* was published in 2016 on “Microbial ecotoxicology: an emerging discipline facing contemporary environmental threats” [33]. Among the excellent articles in this publication are two that speak to different applications of microorganisms in ecotoxicology; one is using pollution-induced community tolerance (PICT) as a tool for monitoring lake restoration [34], and the other is using the bioluminescent characteristics of immobilized *Aliivibrio fischeri* ATCC^®^ 49387^TM^ as a toxicity biosensor [35]. This latter approach has of course been around for a long time and is perhaps best associated with the Microtox^®^ toxicity test developed by AZUR Environmental in 1979 to detect toxicity in water and wastewater. Presence of toxins (to the bacteria) is relatively reliably determined through reduction in bioluminescence [36] in the Microtox^®^ test, measured simply as a reduction in light emission. The test is now an ASTM Standard method (D5660-96, 2009) [37].

The biosensor approach as a microbial ecotoxicology tool is interesting and extends the multiple uses of biosensors in biomedical, environmental and many other applications [38]. Halmi [39] provides a good review on “rapid ecotoxicological tests using bioassay systems”. Later researchers have made improvements in the technology [40,41,42], and I am sure they will continue to do so. An alternative biosensor approach uses a packed bed bioreactor with immobilized consortia of bacteria and monitors respiration in response to toxic metals [43]. While these, and other biosensor technologies, are promising, it is difficult to know how long it will be before they can be used reliably and routinely to monitor ecosystem and environmental health.

## 3. The Omics Age

Our 2005 paper [23] reviewed a number of our NBH studies, including our use of more traditional ecotoxicological tools used in the “RAMP” or Rapid Assessment of Marine Pollution approach [44,45]. Our paper concluded:

“Our long-term goal is to develop multiple probes to evaluate ecological health in marine ecosystems. Our approach will be to develop multiple FISH probes (or micro-arrays) to rapidly hybridize genes that are actively expressed in response to contaminant stress. These biomarkers should correlate with stress (biomarker) responses in higher organisms” [23].

An ideal and lofty goal that of course we did not meet; it therefore becomes of interest to see if other research groups have made further progress. Before we do, it is instructive that in the early 2000s, there was considerable excitement around microarray technologies and the potential to have an array “light up” in response to specific microbial responses to environmental contaminants, but progress in this area turned out to be very slow, due in part we suspect to the ease of whole-genome sequencing. The gene array is not dead, but we are not sure that it is close to realizing its early promise in microbial ecotoxicology. An interesting 2021 paper used functional gene arrays to analyze microbial communities on microplastics [46]. This work used a “new generation” of functional gene arrays that was developed by Shi et al. [47], called GeoChip 5.0. The smaller format of these chips 5.0S was used in the microplastics paper, a format that covers genes related to biogeochemical cycling and others.

Together with 16sRNA sequencing, the authors presented gene expression profiles related to key metabolic processes that differed between microplastic communities and the surrounding waters. This is interesting and promising research, but, as with studies throughout the ages, concludes with the need for more research. This is just a starting point, and I suspect more research with the larger format GeoChip 5.0M would provide stronger information, as its functional categories include biogeochemical cycling of “various metals, stress response, microbial defense, electron transport, plant growth promotion, virulence, *gyrB*, and fungus-, protozoan-, and virus-specific genes” [47]. That said, a search of the literature seems to suggest that GeoChip 5.0M has mainly been used to date for soil biogeochemistry and functional diversity studies [48,49], including tracking virulence and antibiotic resistance genes in manure-amended soils [50,51]. While no direct applications of this technology in microbial ecotoxicology are apparent in a search of the literature, the potential is there. A recent article describes the use of GeoChip 5.0 to demonstrate functional genes in heavy metal-contaminated sediments, including “metal-resistant and organic remediation genes” [52]. It is important to note that these genes were associated with microbial consortia that were added to the sediments to promote bioremediation and not the indigenous microbial communities. The potential for these chips to begin to meet our early expectations in microbial ecotoxicology seems vast. The authors claim “161,961 oligonucleotide probes covering >365,000 genes” and the functional categories mentioned above [47]. We would hope that this provides a degree of resolution that can really help determine stress responses in relation to specific contaminants that could inform remediation.

What has probably slowed down the development of gene chips in microbial ecotoxicology, and indeed most earlier molecular approaches, is of course high-throughput sequencing and the emergence of an array of “omics” used to characterize microbial communities (Table 1). In fact, one publication states, “The future of soil microbiology research is expected to be shaped by the continuous development of metagenomics” [53].

**Table 1:** A brief summary of some of the main “omics” approaches, all of which are worth considering as tools in microbial ecotoxicology, especially “meta” versions that look at community/environmental samples. (HTS—high throughput sequencing; NGS—Next-generation sequencing; NMR—nuclear magnetic resonance spectroscopy).

Omics *	Brief Description	Examples of High-Cost Instrumentation	Opportunities to Reduce Cost and Field Application
Genomics	Study of the gene: probably the first “omics” to emerge in microbial ecology, with the ability to examine presence of specific genes (i.e., metal resistance) to metagenomics (community DNA) [54].	(HTS)/(NGS)	Nanopore sequencing [55]; Bento Lab portable PCR Station [56]; Microarrays **
Transcript-omics	The study of all the RNA transcripts that are produced from the genome. Can reflect, for example, up- or down-regulation of transcription in relation to chemical exposures [57].	RNA reverse-transcribed to cDNA followed by HTS/NGS	Nanopore sequencing; Bento Lab portable PCR Station
Proteomics	Study of protein expression: provides characterization of final gene products, including posttranslational modifications which could be used in ecotoxicology [58].	Mass spectrometry; liquid chromatography	Sensor technologies, i.e., sensor-arrays [59]
Metabolomics	Study of metabolites—can be used to look at metabolic changes as a result of toxic chemical exposure or other stressors [60].	Mass spectrometry; NMR	Sensor technologies—i.e., single cell metabolomics [61]
Volatilomics	The study of volatile metabolites ***. A largely untapped method for characterizing microbial communities [62,63].	Mass spectrometry; NMR and others	Sensor technologies—as used in the food industry [64]

* For all omics, in addition to expense, the greatest challenges are understanding bias and interpretation of extremely complex data. However, bioinformatics and AI are unlocking at least some of the complexity [65]. ** Not cheap at this point, but with great potential. If I were starting again (and had far younger eyes), I would be looking at miniaturized lateral flow assays (“lab-on-a-chip”) solutions for isothermal amplification [66] and microarray studies [67]. *** We usually think of volatile organic compounds (VOCs), but there are volatile inorganics that could prove instructive in ecotoxicology, e.g., methylmercury [68].

We have used metagenomics to characterize surface water microbial communities (and pathogens) in fresh waters [69,70] and the mobile antibiotic and heavy metal resistome in wastewater [71]. These tools are elegant and constantly evolving, and most microbial ecotoxicology today focuses on them, although other fingerprinting methodologies, such as Denaturing gradient gel electrophoresis (DGGE), continue to yield insights [72]. Omics approaches provide an enormous amount of data to build diversity indices and compare sites [73,74], and bioinformatics tools necessary to analyze these vast amounts of data are constantly evolving [75]. It is interesting to see in the literature that there is even a paper on volatilomics in microbial ecotoxicology [62]. These authors looked at volatile organic compounds produced by bacteria in response to a chemical stressor, in this case, pesticides. They conclude that certain VOCs “are probably biomarkers of exposure” and that further investigation is warranted, although there do not appear to be further publications to date from this group using bacteria.

This article is not meant as a scoping review [76]; there are excellent reviews on the state of microbial ecotoxicology to draw upon [77], and even an edited book of that title [78], although, with an emerging field, it is hard to remain current. In terms of omics, an excellent review has been written on use of “genomics, metagenomics, transcriptomics, proteomics, metabolomics, and multi-omics” and their applications to freshwater biomonitoring, using both microbial communities and higher organisms [79]. The situation is clear—while higher organisms remain useful in biomonitoring, the microbial community is still too complex to routinely and reliably use. This can in part be credited to our lack of understanding of “microbial community dynamics”, which may need to be the primary focus moving forward [80].

Other concerns about omics in routine monitoring are the cost of instrumentation, inherent biases in the methodologies and missing data—not all biomolecules are sampled. While these latter problems may eventually be addressed through bioinformatics and AI, routine application would be very much for the future [81]. The cost of instrumentation and the ease of use for field application is in part beginning to be addressed by the Oxford Nanopore Technologies (ONT) MinION [82]. This handheld device can be operated from a laptop computer or similar device and has been deployed at a variety of remote sites, including rainforests [83], Antarctica [84] and the International Space Station [85]. However, it is in routine monitoring of the environment where the MinION can really demonstrate its capacity for rapid, actionable data [70,71,86,87,88] that could be applied in the field of ecotoxicology [89] and may be particularly powerful in combination with stable isotope probing [90].

French researchers have taken a lead in establishing the International Network on Microbial Ecotoxicology, or EcotoxicoMic. This has led to four international conferences, held in 2017, 2020 (virtual), 2022 and 2024. These conferences have directly or indirectly resulted in a number of publications and special issues [91,92,93], which all point to the challenges that need to be surmounted before we have reliable tools. These challenges include the vast diversity of microbes, microbial responses and adaptations, the exposure to multiple stressors and many other challenges—in other words, complexity. The 2024 conference, held in November 2024, has announced a thematic issue in *FEMS Microbiology Ecology* on microbial ecotoxicology, with final submissions due at the end of May 2025 [94].

A key recommendation is not to give up! In many ways, the further development of platforms such as GeoChip 5.0 make the most sense—and the many other types of biological and chemical sensors. Incorporation of these sensors has been discussed for a number of years for ocean monitoring [95,96,97]. The focus for microbial ecotoxicology would be on creating something that was rugged enough to deploy on observing platforms in oceans and other harsh environments and could be easily read and interpreted remotely.

On the “omics” front, low-cost, portable solutions will be important for early warning systems, which are really the goal for microbial ecotoxicology. In my view, sophisticated instrumentation rather misses the point, although it is critical to follow up an initial warning—whether from a sensor or hand-held “omics” device. As stated earlier, metagenomics has been conducted in the field using the ONT MinION. While detection of gene expression in the field remains challenging, there is a large ONT community with an increasing focus on direct RNA sequencing [98]. While I am not aware of applications specific to microbial ecotoxicology, I believe this could be a fruitful area of research.

## 4. Conclusions

40 years on, and on a personal note, I am surprised we are still so far from the routine use of microbiology in ecotoxicology (not counting Microtox^®^ and other basic screening tests [99]). A lack of funding in this area is of course an easy target for blame. I am not unbiased in this, as most of my proposals on this topic—spanning many years—have been rejected! Unfortunately, at least in the USA, the next four years will be a wasteland for environmental research funding, with, I suspect, exponential increases in future needs. The American people have elected someone who will do untold damage to the environment, not just through his “drill, baby, drill” agenda, but through his plans to reverse decades of environmental legislation. We already know the damage that oil and other extractive industries have done to our surface, ground and marine waters, and the need for early warning systems for destabilized ecosystems has never been so great.

The direction that America has taken is depressing beyond belief, but I will try to end on a positive note. If EcotoxicoMic can maintain their initial enthusiasm and their network begin to publish real advances in our “omics” methodologies, then we may make progress. I do look forward to seeing the next thematic issue in *FEMS Microbiology Ecology* on Microbial Ecotoxicology and will hope to see applications and not just basic research.

## Data Availability

No new data were created or analyzed in this opinion piece. Data sharing is not applicable to this article.
